# Oil Palm Frond Juice as Future Fermentation Substrate: A Feasibility Study

**DOI:** 10.1155/2014/465270

**Published:** 2014-06-23

**Authors:** Che Mohd Hakiman Che Maail, Hidayah Ariffin, Mohd Ali Hassan, Umi Kalsom Md Shah, Yoshihito Shirai

**Affiliations:** ^1^Department of Bioprocess Technology, Faculty of Biotechnology and Biomolecular Sciences, Universiti Putra Malaysia (UPM), 43400 Serdang, Selangor, Malaysia; ^2^Graduate School of Life Science and System Engineering, Kyushu Institute of Technology, Fukuoka, Japan

## Abstract

Oil palm frond (OPF) juice is a potential industrial fermentation substrate as it has high sugars content and the OPF are readily available daily. However, maximum sugars yield and storage stability of the OPF juice are yet to be determined. This study was conducted to determine the effect of physical pretreatment and storage duration of OPF petiole on sugars yield. Storage stability of OPF juice at different storing conditions was also investigated. It was found that OPF petiole squeezed by hydraulic pressing machine gave the highest sugars recovery at almost 40 g/kg, accounting for a recovery yield of 88%. Storage of OPF petiole up to 72 hrs prior to squeezing reduced the free sugars by 11 g/kg. Concentrated OPF juice with 95% water removal had the best storage stability at both 4 and 30°C, when it was stored for 10 days. Moreover, concentrated OPF syrup prepared by thermal processing did not give any Maillard effect on microbial growth. Based on our results, OPF juice meets all the criteria as a good fermentation substrate as it is renewable, consistently available, and easy to be obtained, it does not inhibit microbial growth and product formation, and it contains no impurities.

## 1. Introduction

Starch, lignocellulose, and molasses are some of the popular considerations for fermentation substrates over the past decades. Starch, largely contained in the staple food such as potato, maize, rice, and cassava, was found to be conflicting between food supply and biofuel substrate even though it is abundantly produced around the globe. The substrate merely kept as much as 15 billion gallons as maximum production per year [1]. Lignocellulosic feedstock on the other hand has shortcoming in terms of harvesting, collecting, and delivering though it was rendered as nonfood substrates [1]. Lignocelluloses such as corn stover, rice straw, sugarcane bagasse, and oil palm biomass are produced from agricultural activities and regarded as low value products. Meanwhile, molasses feedstock is found to be tangled between the food crisis and high value products manufacturing. It is considered as byproduct of sugar refining derived from sugarcane, sugar beet, and grapes and also used for animal feed in certain countries such as USA, Japan, the Netherlands, and UK [2]. With all the issues related to food scarcity and animal feed, these substrates open many challenges for the fermentation industry and have unwrapped another possibility for another undeveloped feedstock substrate such as oil palm frond (OPF) juice.

Being one of the largest palm oil producers in the world, Malaysia generated approximately 80 million tonnes of dry solid biomass from the oil palm industry in 2010. This figure is expected to reach up to 110 million tonnes in the year 2020 [3]. The major part of the solid biomass deposited on the plantation was found to be OPF which currently contributes to nearly 60% of the volume. Naturally, fronds are left at the plantation during pruning and harvesting in order to ensure the nutrient conservation in the soil. This is due to the current belief that OPF is beneficial for nutrient recycling and soil conservation [4–6]. Nevertheless, there have been some reports on the potential utilization of OPF fiber for animal feeds [6], paper and pulp [7, 8], polymer composite, [9] and nutraceuticals [10, 11]. Recently, our group reported on the possibility of obtaining OPF juice by simply pressing the OPF petiole. OPF juice contains high amount of sugars, making it a potential fermentation feedstock for various value-added products such as polyhydroxyalkanoates (PHA), bioethanol, biobutanol, lactic acid, and succinic acid.

The basal part of the OPF petiole which is 1/3 of its length houses 66% of the total sugars in OPF, while the nutrients mostly centered at the middle and top parts of the fronds [3]. Therefore, it is expected that utilizing the basal portion of the OPF for industrial fermentation will not interfere with the nutrients supply to the soil. Furthermore, abundant supply of OPF petioles is guaranteed from plantation because fronds are constantly available during fronds pruning for fruit harvesting.

In order to be a good industrial fermentation feedstock, there are some criteria that need to be fulfilled. The substrate needs to be cost practical, to produce high yield of biomass and product of interest, to be consistently and locally available, to be easily operated, to contain no impurities, to have low risk on health and safety, and to meet the local government legislation. Previous study has shown that OPF juice is suitable to be used as fermentation feedstock as there was no inhibition on microbial growth or product formation, there were no impurities, it was easy to be operated, and it had no risk on health and safety [12]. The use of OPF juice as renewable fermentation feedstock should be of industrial interest as OPF is readily available all year round, no enzymatic or chemical pretreatment will be needed unlike lignocellulosic materials, and, most importantly, there are no inhibitors or salts that will affect the fermentation yield. Salts and inhibitors like weak acids, furan derivatives, and phenolic compounds which are produced during steam pretreatment and hydrolysis of lignocellulose materials may affect the performance of product-generating microbes [13, 14].

Industrially viable fermentation substrate should take into account the easiness to obtain the substrate and the recovery yield of the substrate during processing step. OPF juice has been proven to be a good fermentation substrate for lab-scale PHA production [12]. This study is hence made available to provide the information on the effect of shredding and pressing method on the recovery of sugars from OPF petiole. Furthermore, the effect of OPF petiole storage prior to juice extraction was also studied in order to stipulate the appropriate storage duration of the OPF petiole. Moisture escape and microbial attack during storage might affect free sugars recovery and hence the storage evaluation study is crucial. The report in this study would be helpful to disclose the information on degradation study of OPF juice and a handy approach to delay the sugar loss. Similar study has never been reported elsewhere; therefore, this study is needed to estimate the yield of sugars per kg of OPF and its storage stability.

## 2. Materials and Methods

### 2.1. Raw Material Preparation

**Figure 1:**
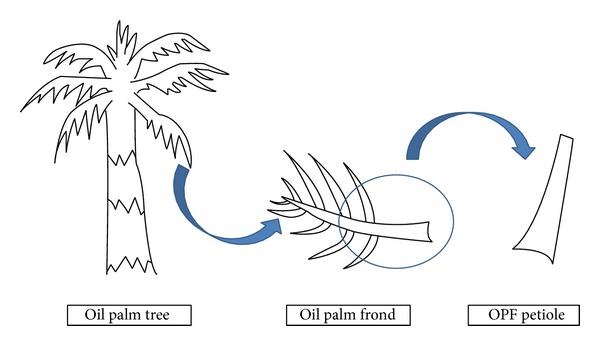
Chronology of OPF petiole collection from the oil palm plantation. Only basal part of the petiole was collected, which is about 1/3 of the original petiole length.

Fresh OPF petioles were obtained from Ladang Kelapa Sawit 6 Hektar Universiti Putra Malaysia (UPM). The OPF petiole has an average length of 2-3 m, and in this study only the basal part was used, which is approximately 1/3 of the original OPF length. The weight of the OPF used in this study was between 2 and 2.5 kg. Freshness of harvested petiole was kept assured by collecting the petioles and processing them on the same day. Delay between transportation and processing was about 1 to 1.5 hours. The fronds were also pruned at both edges before processing to remove dirt. All petioles were kept in sealed plastic bags at ambient temperature (28–30°C) prior to pressing; Figure 1 illustrates the chronology of OPF petiole collection.

### 2.2. Determination of Potential Total Free Sugars in OPF Petiole

Fresh petiole weighing 200 g was blended using Waring commercial blender model 32BL80. 100 mL water was added during blending. Juice was wrung out of the crushed OPF after blending. This step was repeated five times. Extracted juice was then filtered with 0.2 *μ*m Whatman membrane nylon filter before undergoing HPLC analysis for sugar content determination.

### 2.3. OPF Juice Extraction

Extraction of OPF juice from OPF petiole was done by using a pilot scale hydraulic pressing machine (MATSUO Inc., Japan) at Forest Research Institute of Malaysia (FRIM). Pressure was set at 30 MPa. Subsequently, obtained juice was filtered to remove fibrous solids and scum. The juice was further clarified by withdrawing solid particles using centrifuge operated at 15000 ×g for 15 minutes at 4°C (Thermo Fisher Scientific, NC, USA). The filtrate was stored at −20°C before sugar analysis [12].

In order to determine the effect of shredding, OPF petiole was shredded using a pilot-scale shredder (Matsuo Inc., Japan) prior to pressing.

### 2.4. Effect of OPF Petiole Storage Duration

Effect of OPF petiole storage duration on the recovery of sugars was determined in this study. Fresh OPF petioles harvested from the plantation were stored under shaded area at ambient temperature (28−30°C) and relative humidity (75–95%). Samples were taken every 24 hours for 72 hours and were checked for weight reduction, moisture content, and sugar recovery during pressing. In this study, pressing was done by conventional sugar cane pressing machine (Elephant, W.H.L. Machinery, Malaysia).

### 2.5. OPF Juice Storage Stability

Effect of storage temperature and water removal on the stability of sugars in OPF juice was evaluated by determining sugars content and microbial count before and after storage.

#### 2.5.1. Effect of Storage Temperature

Effect of storage temperature was studied by storing the OPF juice at 4 and 30°C. Storage study was conducted up to 10 days and sample was collected at 24-hour intervals for sugar content analysis.

#### 2.5.2. Effect of Water Removal and Water Activity

Effect of water removal and consequently water activity on storage stability of OPF juice was conducted by concentrating the OPF juice using two methods: direct boiling and vacuum evaporation by laboratory scale rotary evaporator (Rotavapor R-210/R-215, Büchi, Switzerland). Water removal was set at 50, 60, 70, 80, 90, and 95% from its original water content (93.8%). For direct boiling, OPF juice was heated on heat stirrer at 98°C, while, for vacuum evaporation, OPF juice was heated in a rotary vacuum evaporator operated at 55°C. Concentrated samples were then stored for 10 days. Sampling was done at 24-hour intervals for sugar content determination.

### 2.6. Effect of OPF Juice Heating on Microbial Growth

Heating sugars in the presence of amino acid may cause browning due to Maillard reaction [15]. Effect of heating on the microbial growth was tested in order to confirm the suitability of the concentrated OPF juice for fermentation substrate. Several microorganisms commonly used for fermentation were tested for their microbial growth on heat-treated OPF juice such as* Bacillus cereus, Bacillus subtilis, Escherichia coli, Staphylococcus aureus, Saccharomyces cerevisiae, Mucor, Penicillium*, and* Trichoderma*. For the microbial growth testing, filter-sterile concentrated OPF juice was mixed with autoclaved plain agar without any additional nutrients. Control medium was prepared with synthetic sugars mimicking sugar content in OPF juice. All microorganisms were cultured and incubated at their respective optimum growth temperature.

### 2.7. Analyses

#### 2.7.1. Sugar Content by HPLC

Sugar content in the OPF juice was analyzed using HPLC (Shimadzu HPLC LC-20A, Japan) with an RI detector. The HPLC column (Phenomenex, LT Resources, Malaysia) used was Rezex RCM Monosaccharide Ca^2+^ (8%) (300 × 75 mm) operated at 80°C. The mobile phase was 100% water at a flow rate of 0.6 mL/min. Calibration curves were made for individual sugar, using commercial merchandise purchased from Sigma-Aldrich Chemical (Malaysia). Each of the sugar components was determined by linking the retention times with those of reliable standards and quantified by external standard scheme [16].

#### 2.7.2. Moisture Content

For moisture content determination, ground OPF petiole was dried in heating oven at 105°C for 48 hours. The fiber was weighed every 24 hours until constant weight was achieved.

#### 2.7.3. Water Activity

Water activity for untreated and treated OPF juice samples was tested using water activity meter (FA-st Lab, California) operated at 28°C and 1 atm. Samples undergoing this test were freshly prepared and tested on the same day.

#### 2.7.4. Microbial Count

Microbial count testing was conducted by using agar plate count technique. Concentrated OPF juice samples with 50, 60, 70, 80, 90, and 95% water removal were prepared fresh for the microbial count test. 100 *μ*L of diluted samples was pipetted onto the agar and incubated at 30°C for 48 hours. Cell colony was counted after 48-hour incubation.

## 3. Results and Discussion

### 3.1. Total Free Sugars in OPF Petiole

**Figure 2:**
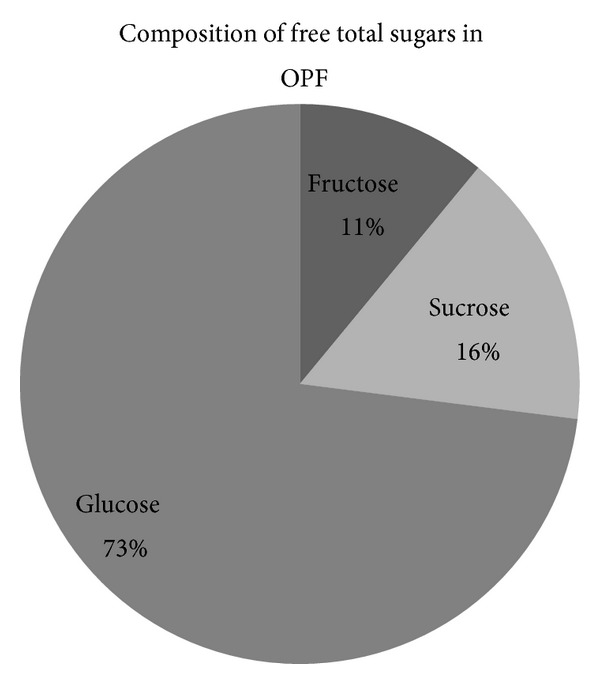
Proportion of free sugars in OPF juice extracts.

Free sugars content in OPF petiole has never been reported before. In this study, total free sugars (glucose, fructose, and sucrose) content in the basal part of OPF petiole was determined by grinding and wringing the OPF petiole in order to get the juice, followed by sugars analysis by HPLC. It was found that potential free sugar content in OPF petiole was 44.8 g/kg. The composition of free sugars (%) in OPF petiole is represented in Figure 2.

### 3.2. Effect of Physical Pretreatment on the Recovery of Sugars from OPF Petiole

**Figure 3:**
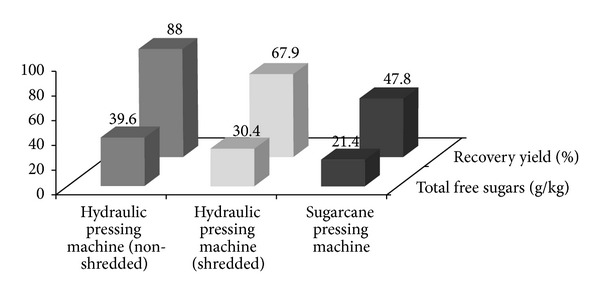
Yield of sugars obtained by using conventional sugar cane pressing machine and hydraulic pressing machine (with and without shredding).

Two different pressing machines were used for extracting the juice, that is, conventional sugar cane pressing machine and hydraulic pressing machine.  Figure 3 shows sugar recovery from the two different pressing machines. Approximately 39.6 g/kg of sugars can be recovered using hydraulic pressing machine, rendering sugar recovery of 88% from the total potential free sugars inside OPF petiole. On the other hand, sugar cane pressing machine gave sugars recovery of only 21.4 g/kg, or 47.8% of the total potential free sugars in OPF petiole. The difference in the sugars recovery is due to the difference in the mechanism of pressing by the two pressing machines. Hydraulic pressing machine is equipped with hydraulic pressurizing system which provides better pressing effect, and, hence, more sugars could be recovered.

**Figure 4:**
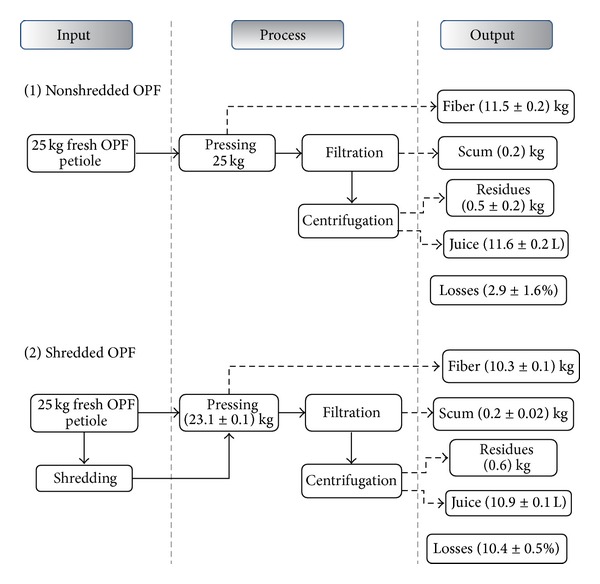
Mass balance of OPF juice extraction from OPF petiole for nonshredded and shredded OPF. Specific gravity of OPF juice in this study was 1.04 ± 0.004.

Effect of shredding on the recovery of sugars from OPF petiole was later conducted. Both shredded and nonshredded OPF petioles were pressed using hydraulic pressing machine. It was found that shredding did not give better effect on the sugars recovery. Sugars recovery was lower for shredded OPF petiole, and this was contrary to our hypothesis as we expected that more sugars could be recovered due to increased surface area after shredding which may allow more sugars to come out. Based on the mass balance of the two processes (Figure 4), loss of mass was higher for shredded OPF compared to the nonshredded OPF. The difference was about 7.5%. Higher loss for shredded OPF was due to the shredding step, whereby almost 2 kg of OPF petiole fiber was lost due to being blown off during shredding. The loss during shredding caused the overall sugars recovery for shredded OPF to be lower compared to the nonshredded OPF.

### 3.3. Evaluation of Appropriate OPF Petiole Storage Period prior to Juice Extraction

**Figure 5:**
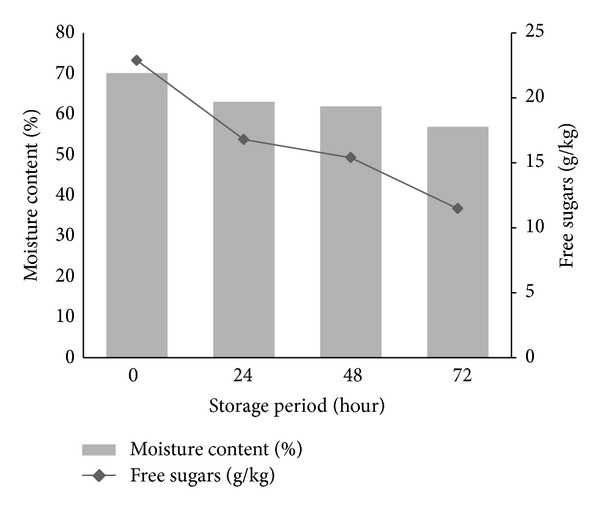
Effect of storage period on the OPF petiole moisture content and total free sugars in OPF juice. Results are means ± SD of three determinations.

It is important to contemplate the storage period of OPF petiole prior to OPF juice extraction as storing the OPF petiole at ambient condition may cause reduced recovery of sugars due to free sugars degradation by microorganisms and also reduced moisture content which makes pressing process difficult. From Figure 5, it is seen that both moisture content and sugars recovery showed similar profiles, whereby the trend was declining with the increase in storage period. During 72 hours of storage, there was a substantial drop in moisture content of OPF petiole, juice yield, and total sugars recovery. Initial value of recovered sugars in the OPF petiole was 22.9 g/kg. Within 24 hours, moisture escaped as high as 7% giving the overall yield of sugars extracted a fall to 16.8 g/kg. Sugars gradually dropped to 15.4 g/kg and finally 11.5 g/kg for 48 and 72 hours of storage, respectively. Until 72 hours, the overall losses halved from the initial value, let alone the moisture escaped which accounted up to 13% reduction. Both the juice quantity and quality (sugar content) reduced within 4 days of storage suggested that the collected OPF petioles need to be pressed immediately after they are pruned.

It has been reported earlier that storing oil palm trunk (OPT) prior to pressing will increase sugars content in the OPT sap [17]. The contrary finding herein can be explained by the difference in the physical characteristics of OPF and OPT. OPF tends to lose moisture easily due to its thinner skin and smaller size. The conditions also make OPF more prone to microbial attack which caused depletion of sugar content.

### 3.4. OPF Juice Storage Stability

Tables 1(a) and 1(b) demonstrate sugar loss after 10 days of storage and microbial count (CFU/mL) for (a) raw and (b) concentrated OPF juice stored at 4 and 30°C. Two methods were used to concentrate the OPF juice: direct boiling and vacuum evaporation. Overall, direct boiling of OPF juice followed by storing at 4°C resulted in the least sugar degradation. Effect of water removal on sugar loss can be clearly seen from the table. Raw OPF juice recorded the highest sugar loss followed by concentrated OPF juice at water removal of 60, 80, 90, and 95%. Meanwhile, effect of storage temperature was pronounced for lower water removal (60 and 80%). At higher water removal (90 and 95%), storage temperature did not seem to affect sugar loss very much.

Both direct boiling and vacuum evaporation methods gave almost similar results in terms of total sugar loss and microbial count, indicating that method for concentrating OPF juice did not give marked effect on the OPF juice. Number of viable microorganisms (CFU/mL) after 10 days of storage was the highest for raw OPF juice, followed by concentrated OPF juice with 60, 80, 90, and 95% water removal. Lower number of viable microorganisms in concentrated OPF juice could be due to the effect of heat treatment applied on OPF juice.

Another reason for the less number of viable microorganisms in the concentrated OPF juice is due to water activity, *a*_w_ in the concentrated OPF juice.  Table 2 shows *a*_w_ values of concentrated OPF juice at different water removal (%). Water activity reduced at higher water removal. This is expected, since water activity is related to the water content in a sample. Low water activity sample leads to the least favourable condition for microorganisms to grow. *a*_w_ of as low as 0.833 was acquired through direct boiling of OPF juice with water removal of 95%. It has been reported that *a*_w_ of 0.87 was pragmatically enough to retard most of food-borne microbes [18].

Typically, low temperature would produce prominent environment for juice storage, but consistent with real application, where transport and storage space is limited, storage at ambient temperature would be the best option. Based on our results, storing OPF juice at temperature 30°C did not reduce sugar content in OPF juice too much, provided the juice is concentrated with 90–95% water removal. Application of temperate heat on the other hand was not a suitable method to preserve the OPF juice, due to the potential of contamination by certain microbes such as* C. botulinum* types B, E, and F that survive under moderate abuse condition [19]. The treatment of OPF juice with higher temperature killed more microbes and hence provided a better environment for sugar stability for prolonged storage.

**Figure 6:**
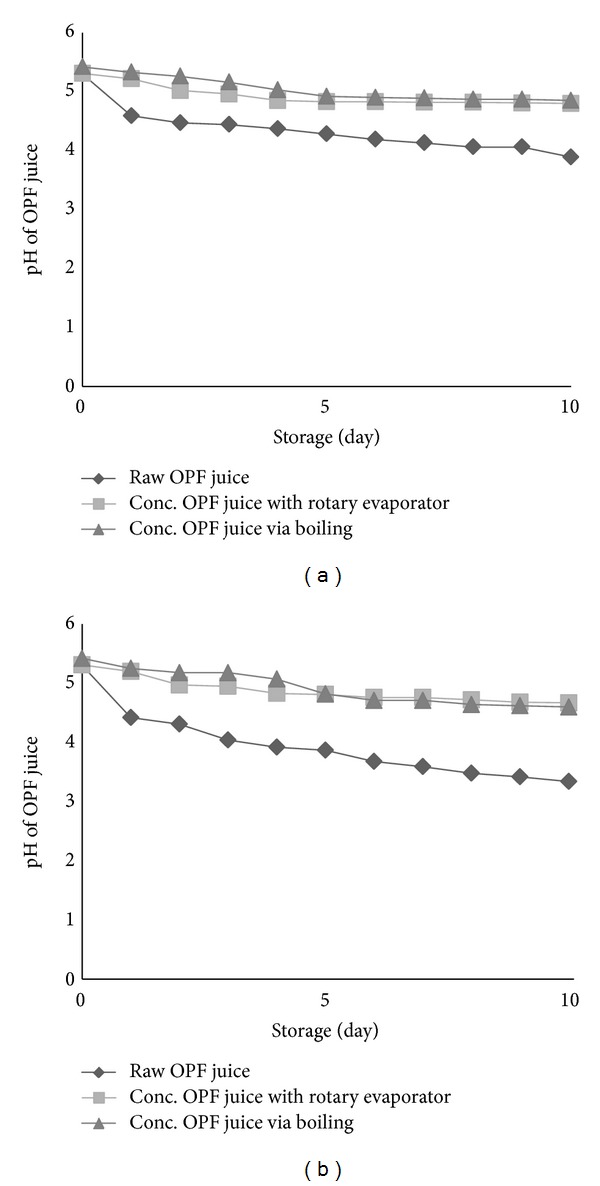
(a) pH profile for concentrated OPF juice through 95% water removal and untreated fresh juice evaluated for 10 days at 4°C. (b) pH profile for concentrated OPF juice through 95% water removal and untreated fresh juice evaluated for 10 days at 30°C. Results are means ± SD of two determinations.

pH analysis is a brief indicator for spoilage of sugary juice. Figures 6(a) and 6(b) show the pH changes with storage time. It was observed that OPF juice deterioration started almost immediately for unconcentrated juice (raw juice). The pH was dramatically decreased after the first day of storage and it started to decelerate from the second day onwards for both storage temperatures of 4 and 30°C. However, pH drop was higher for raw OPF juice stored at 30°C. In the case of concentrated OPF juice (95% water removal), pH drop was very little and can almost be neglected. Results in Figures 6(a) and 6(b) are consistent with total sugar loss in OPF juice (Table 1), whereby the higher the total sugar loss, the lower the pH of the juice. This can be explained by the metabolism activity of some microorganisms which will convert sugars to acid in favourable condition. In raw OPF juice, number of viable microorganisms was much higher and, therefore, pH dropped drastically compared to concentrated OPF juice.

### 3.5. Effect of OPF Juice Heating on Microbial Growth

Since concentrated OPF juice was prepared by heat treatment, there is necessity to check for the potential of Maillard reaction as OPF juice contains amino acids [12] which may probably react with reducing sugars during heating at high temperature.  Table 3 shows the ability of several microorganisms usually used in fermentation to grow on medium containing concentrated OPF juice. Based on our observation,* Bacillus cereus* and* Bacillus subtilis* were found tolerant and were successively grown on medium containing boiled OPF juice. Successive growth of these two most vulnerable strains [20] suggested that heating the OPF juice did not contribute to the Maillard reaction and, consequently, there was no inhibition of microbial growth. Other microorganisms tested in this study also showed positive growth showing that the methods used for concentration of OPF juice did not affect the juice and, hence, are suitable and could be applied for real application. The ability to use direct boiling for concentrating and preserving OPF juice is an advantage especially for industrial application as there will be no special equipment or condition which would be required for the purpose.

## 4. Conclusions

OPF juice recovery was mainly dependent on time lapsing between OPF harvesting and pressing. Short time lapse would avoid the depletion in juice yield. OPF petiole pressed using hydraulic pressing machine gave the best sugars recovery at 39 g/kg, which contributed to 88% of recovery yield. Shredding OPF juice prior to pressing was found not effective in enhancing sugars recovery. This is due to loss of OPF mass and moisture during shredding process. Concentrating OPF juice up to 95% water removal ensures sugar stability in OPF juice even at temperature of 30°C. Sugar loss recorded for OPF juice with 95% water removal was only 6–8%, after storing for 10 days. It was also found that both methods used for water removal in this study, that is, vacuum evaporation and direct boiling, did not affect OPF juice composition and did not produce Maillard effect, making the methods suitable for concentrating OPF juice. Overall, it is suggested that OPF juice could be a potential renewable substrate for fermentation process considering that the free sugars are obtainable with a simple mechanical treatment, are consistently available since OPF is pruned daily, do not inhibit microbial growth, and contain no impurities.

## Figures and Tables

**(a):** 

	Total sugar loss after 10 days (%)		Microbial count after 10 days (CFU/mL)	
	Stored at 4°C	Stored at 30°C	Stored at 4°C	Stored at 30°C
Raw OPF juice	29.5	56.5	7.2 × 10^6^	5.0 × 10^7^

Results are means ± SD of two determinations.

**(b):** 

Water removal (%)	Rotary evaporator			Direct boiling		
	Total sugar loss after 10 days (%)	Microbial count (CFU/mL)		Total sugar loss after 10 days (%)	Microbial count (CFU/mL)	
		Day 0	Day 10		0th day	10th day
Storage at 4°C						
60	32.1	3.0 × 10^4^	2.7 × 10^6^	27.3	3.0 × 10^4^	2.1 × 10^6^
80	18.9	2.0 × 10^4^	8.5 × 10^5^	16.8	2.0 × 10^4^	4.7 × 10^5^
90	12.8	8.5 × 10^3^	4.0 × 10^5^	13.7	8.5 × 10^3^	2.5 × 10^5^
95	8.1	4.5 × 10^3^	3.0 × 10^5^	6.3	4.5 × 10^3^	2.0 × 10^5^
Storage at 30°C						
60	37.7	3.8 × 10^4^	3.5 × 10^6^	35.2	3.0 × 10^4^	2.0 × 10^6^
80	19.2	2.4 × 10^4^	1.1 × 10^6^	18.1	2.0 × 10^4^	5.5 × 10^5^
90	12.9	2.0 × 10^4^	5.6 × 10^5^	13.2	8.5 × 10^3^	2.5 × 10^5^
95	8.5	1.5 × 10^4^	3.0 × 10^5^	10.5	4.5 × 10^3^	2.5 × 10^5^

Results are means ± SD of two determinations.

**Table 2:** Water activity of concentrated OPF juice.

Water removal (%)	*a*_w_ at 28°C	
	Direct boiling	Rotary evaporator
60	0.937	0.937
80	0.923	0.925
90	0.886	0.887
95	0.833	0.836

**Table 3:** Microbial growth test on untreated and concentrated OPF juice (water removal of 95%).

Microorganisms	Treated OPF juice		
	Control	Boiling (95% moisture removal)	Rotary evaporator (95% moisture removal)
*Bacillus cereus *	*✓*	*✓*	*✓*
*Bacillus subtilis *	*✓*	*✓*	*✓*
*Escherichia coli *	*✓*	*✓*	*✓*
*Staphylococcus aureus *	*✓*	*✓*	*✓*
*Saccharomyces cerevisiae *	*✓*	*✓*	*✓*
*Mucor *	*✓*	*✓*	*✓*
*Penicillium *	*✓*	*✓*	*✓*
*Trichoderma *	*✓*	*✓*	*✓*

## References

[1] Alternative fuel data centre US Department of Energy. http://www.afdc.energy.gov/fuels/ethanol_feedstocks.html.

[2] Paturau JM Alternative uses of sugarcane and its by-products in agroindustries. http://www.fao.org/docrep/003/s8850e/s8850e03.htm.

[3] Agensi Inovasi Malaysia National Biomass Strategy 2020: New wealth creation for Malaysia’s palm oil industry.

[4] Hassan OA, Ishida M, Shukri IM, Tajuddin ZA Oil palm fronds as a roughage feed source for ruminants in Malaysia oil-palm fronds as a roughage.

[5] Wan Zahari M, Abu Hassan O, Wong HK, Liang JB (2003). Utilization of oil palm frond—based diets for beef and dairy production in Malaysia. *Asian-Australasian Journal of Animal Sciences*.

[6] Zahari MW, Alimon AR, Wong HK Utilization of oil palm co-products as feeds for livestock in Malaysia.

[7] Wanrosli WD, Zainuddin Z, Law KN, Asro R (2007). Pulp from oil palm fronds by chemical processes. *Industrial Crops and Products*.

[8] Yakari MI (2008). *Oil Palm Frond (OPF) as an Alternative Source of Pulp Production Material*.

[9] Isa M, Jidin M, Rahman WA, Aizan W, Palm O Oil Palm Frond ( OPF ) or Coir Fiber ( CF ): Effect of Particle Sizes on the Tensile Properties and Morphology of Natural Fiber Reinforced HDPE.

[10] Abeywardena M, Runnie I, Nizar M, Suhaila M, Head R (2002). Polyphenol-enriched extract of oil palm fronds (Elaeis guineensis) promotes vascular relaxation via endothelium-dependent mechanisms. *Asia Pacific journal of clinical nutrition*.

[11] Jaffri JMD (2009). *Effects of Palm Frond Metahnolic Extract on Blood Pressure, Antioxidant Status and Selected Organs of Nitric Oxide-Deficient Rats*.

[12] Zahari MAKM, Zakaria MR, Ariffin H (2012). Renewable sugars from oil palm frond juice as an alternative novel fermentation feedstock for value-added products. *Bioresource Technology*.

[13] Palmqvist E, Hahn-Hägerdal B (2000). Fermentation of lignocellulosic hydrolysates. I: inhibition and detoxification. *Bioresource Technology*.

[14] Rumbold K, van Buijsen HJJ, Gray VM (2010). Microbial renewable feedstock utilization: a substrate-oriented approach. *Bioengineered Bugs*.

[15] Liu J, Ru Q, Ding Y (2012). Glycation a promising method for food protein modification: physicochemical properties and structure, a review. *Food Research International*.

[16] Kafkas E, Koşar M, Türemiş N, Başer KHC (2006). Analysis of sugars, organic acids and vitamin C contents of blackberry genotypes from Turkey. *Food Chemistry*.

[17] Yamada H, Tanaka R, Sulaiman O (2010). Old oil palm trunk: a promising source of sugars for bioethanol production. *Biomass and Bioenergy*.

[18] U.S. Food and Drug Administration (2011). *Chapter 13: Clostridium Botulinum Toxin Formation (A Biological Hazard)*.

[19] Beuchat LR (1981). Microbial stability as affected by water activity. *Cereal Foods World*.

[20] Einarsson H, Snygg BG, Eriksson C (1983). Inhibition of bacterial growth by maillard reaction products. *Journal of Agricultural and Food Chemistry*.

